# Brain Electrodynamic and Hemodynamic Signatures Against Fatigue During Driving

**DOI:** 10.3389/fnins.2018.00181

**Published:** 2018-03-27

**Authors:** Chun-Hsiang Chuang, Zehong Cao, Jung-Tai King, Bing-Syun Wu, Yu-Kai Wang, Chin-Teng Lin

**Affiliations:** ^1^Centre for Artificial Intelligence, Faculty of Engineering and Information Technology, University of Technology Sydney, Ultimo, NSW, Australia; ^2^Brain Research Center, National Chiao Tung University, Hsinchu, Taiwan; ^3^Department of Computer Science and Engineering, National Taiwan Ocean University, Keelung, Taiwan

**Keywords:** EEG, fNIRS, driving, fatigue, fighting

## Abstract

Fatigue is likely to be gradually cumulated in a prolonged and attention-demanding task that may adversely affect task performance. To address the brain dynamics during a driving task, this study recruited 16 subjects to participate in an event-related lane-departure driving experiment. Each subject was instructed to maintain attention and task performance throughout an hour-long driving experiment. The subjects' brain electrodynamics and hemodynamics were simultaneously recorded via 32-channel electroencephalography (EEG) and 8-source/16-detector functional near-infrared spectroscopy (fNIRS). The behavior performance demonstrated that all subjects were able to promptly respond to lane-deviation events, even if the sign of fatigue arose in the brain, which suggests that the subjects were fighting fatigue during the driving experiment. The EEG event-related analysis showed strengthening alpha suppression in the occipital cortex, a common brain region of fatigue. Furthermore, we noted increasing oxygenated hemoglobin (HbO) of the brain to fight driving fatigue in the frontal cortex, primary motor cortex, parieto-occipital cortex and supplementary motor area. In conclusion, the increasing neural activity and cortical activations were aimed at maintaining driving performance when fatigue emerged. The electrodynamic and hemodynamic signatures of fatigue fighting contribute to our understanding of the brain dynamics of driving fatigue and address driving safety issues through the maintenance of attention and behavioral performance.

## Introduction

Driving safety has attracted increasing attention from the general public as a result of the increasing number of road traffic crashes. Risky driving behaviors, such as fatigue performance, boost drivers' risk of crashing, as increasing fatigue leads to the suppression of drivers' performance, including awareness, recognition and direction control of the car (Lee, 2008).

Fatigue has a pervasive influence on human life and may be experienced on a regular basis (Hockey, 2013). The early definitions of fatigue fail to account for the sleepiness of drivers, and a wide range of factors are associated with transport operator exertion, such as motivation and individual, organizational and environmental factors (Phillips, 2015). In fact, fatigue is generally divided into two forms, physical fatigue and mental fatigue (Lal and Craig, 2001). Specifically, physical fatigue refers to a situation in which humans are less likely to maintain behavioral performance as muscular power drains, and it is often accompanied by mental fatigue, which gradually impairs cognitive ability (distraction, frustration, and discomfort) (Taylor and Dorn, 2006). Mental fatigue often arises from a prolonged period of cognitive-demanding tasks, which are influenced by many factors, such as the environment, physical activity and recuperation periods (Philip et al., 2005). The major symptom of mental fatigue is an ordinary consciousness of tiredness, sensing of inhibition and impaired activity (Marcora et al., 2009). The negative effect of mental fatigue is typically related to poor cognitive performances. Experimental evidence indicates that mental fatigue impairs physical performance in humans through a higher perception of effort rather than cardiorespiratory and musculoenergetic mechanisms (Marcora et al., 2009). Among the two forms, mental fatigue is particularly influential in everyday life; however, individuals tend to underestimate the consequences. For example, mental fatigue is regarded as one of the major factors leading to traffic accidents, which cause different levels of injuries, varying from mild flesh wounds to severe fatalities (Ruei-Cheng et al., 2004).

It is important to note that the accumulating number of traffic accidents has aroused a growing concern in modern societies (Armstrong et al., 2008; Borghini et al., 2012). In professional drivers, mental fatigue-related traffic crashes often occur in high-speed driving scenarios. The main reason is that higher levels of driving fatigue may diminish a driver's arousal and information processing abilities in an unusual and emergency situation (Lal and Craig, 2001). The Federal Motor Carrier Safety Administration (FMCSA) has identified three main factors that lead to fatigue in driving tasks: circadian rhythm effects (circadian influences), sleep deprivation (sleep homeostasis factor), and cumulative fatigue effects (nature of task effects) (Williamson et al., 2011). Although these three factors are likely to coexist in an incident, the major research interest, including our study, is to explore the impact of cumulative fatigue effects on driving performance. The cumulative fatigue performance is reflected not only in drivers' behaviors but also in brain dynamics.

Some previous studies have measured behavioral (such as reaction time (RT) and movement) or neurophysiological signals (such as brain wave and heart rate) to qualify fatigue states or the vigilance decrement. They investigated the impact of the monotony of driving performance by steering wheel movements (Thiffault and Bergeron, 2003) and evaluated the differential psychophysiological reactivity of drivers (Mulders et al., 1982). Moreover, the vigilance decrement of drivers was expressed as increased RTs (Pattyn et al., 2008). Another research work reported the identification of fatigue and high strain under a cyclic loading procedure, which has a potential to trigger a switch of control from human to computer through an adaptive automation interface (Hockey et al., 2009).

Electroencephalography (EEG), which features a high temporal resolution, is widely adopted to explore the brain dynamics of electrical activities. Substantial literature has indicated changes of EEG power spectral with respect to cumulative fatigue effects. Previous studies have suggested that dynamic changes of theta, alpha and beta EEG power in the frontal, parietal and occipital cortex were highly consistent with the level of mental fatigue (Boksem et al., 2005). Specifically, previous findings have shown significant increases in delta and theta EEG power, as well as slight increases in alpha and beta EEG power after prolonged driving tasks (Lal and Craig, 2002). The declined theta and enhanced alpha activities in the parietal and occipital areas were highly correlated with deteriorated performance (Klimesch et al., 1997). Moreover, the increased alpha power was identified as the driving error increased (Campagne et al., 2004) or fatigue occurred (Eoh et al., 2005; Otmani et al., 2005; Jap et al., 2009), which suggests that the enhanced alpha power indicates a higher fatigue. In addition, pre-stimulus EEG delta, theta and alpha activities in the parietal and occipital cortices are highly correlated with task performance (Klimesch, 1999).

Brain EEG dynamics contribute to the assessment of fatigue levels, whereas hemodynamic analyses have shown that different levels of fatigue lead to varying brain activations as assessed by functional Magnetic Resonance Imaging (fMRI) measurement, a neuroimaging technique used to detect dynamic variations in cerebral blood flow (CBF, i.e., hemodynamic response) (Lange et al., 2005; Cook et al., 2007). A recent study demonstrated that the blood oxygen level dependent (BOLD) responses of faster RTs were greater than those with slower RTs in the inferior parental cortex when performing a psychomotor vigilance task. Furthermore, CBF may be slower in the inferior frontal and parietal cortex using arterial spin labeling (ASL) perfusion fMRI after a series of mental work (Lim et al., 2010). Nevertheless, the shortage of mobility limits the possibility of fMRI in a driving task that involves natural movements, steering, and kinesthetic feedback. One solution in such scenarios is to apply a new neuroimaging technology—functional near-infrared spectroscopy (fNIRS), featuring high temporal and spatial resolutions (Jasdzewski et al., 2003), which is designed to detect functional imaging of brain activation through monitoring changes in the cerebral concentrations of oxy- and deoxy-genated hemoglobin, denoted as HbO and HbR, respectively (Jasdzewski et al., 2003). Thus, fNIRS can measure brain hemodynamic responses (e.g., BOLD response) associated with neuron behavior (Hoshi, 2003). On the basis of prior studies (Thiffault and Bergeron, 2003; Armstrong et al., 2008; Williamson et al., 2011; Borghini et al., 2012), subjective fatigue was inversely correlated with HbO augmentation over the ventrolateral prefrontal cortex during an executing cognitive task (Mulders et al., 1982). Moreover, recent research described that in a context with insufficient sleep, the activation level of HbO in the frontal lobe declines in cognitive functions (Pattyn et al., 2008) because participants are not prone to recover the normal concentration of HbO from insufficient sleep in a short time.

Multi-modal integration, a hybrid system combining EEG and fNIRS signals, is an attractive approach to investigate drivers' brain dynamics (Ahn et al., 2016), mental stress effects (Al-Shargie et al., 2017), and the further direction of brain-computer interface (BCI) (Ahn and Jun, 2017). A variety of psychophysiological parameters have been regarded as indicators of driving fatigue. For example, recent research showed the evaluation of drivers' mental fatigue by recording EEG and fNIRS simultaneously (Ahn et al., 2016), and the utilization of a combined EEG/fNIRS system to detect driver drowsiness (Nguyen et al., 2017). However, few studies have elaborated the brain electrodynamic and hemodynamic dynamics on how to fight fatigue to maintain attention and behavioral performance during a prolonged driving task. In this study, we expect that participants can steer the vehicle back to the cruising lane as fast as possible to counteract an accumulated fatigue when a deviation occurs. To provide a valuable asset on the brain dynamics of fatigue-fighting driving, the goals of this study are segmented into three parts: (1) Using EEG and fNIR measurements, simultaneously record brain electrodynamics and hemodynamics during a sustained-attention driving task. (2) Identify the association between behavioral performance (RTs) and EEG power spectra. (3) Recognize the hemodynamic signatures to maintain task performance when the EEG-related signatures of fatigue occur.

## Materials and methods

### Participants

In this study, 16 right-handed subjects, aged 22–28 years, participated in a simulated driving experiment. All subjects had normal or corrected-to-normal vision. No subjects reported a history of psychiatric disorders, neurological disease, or drug use disorders. All subjects were recruited from university students and staff at National Chiao Tung University, Taiwan. The Institutional Review Board of National Chiao Tung University approved this study. Informed consent was obtained from all subjects prior to study entry.

### Experimental environment

**Figure 1:**
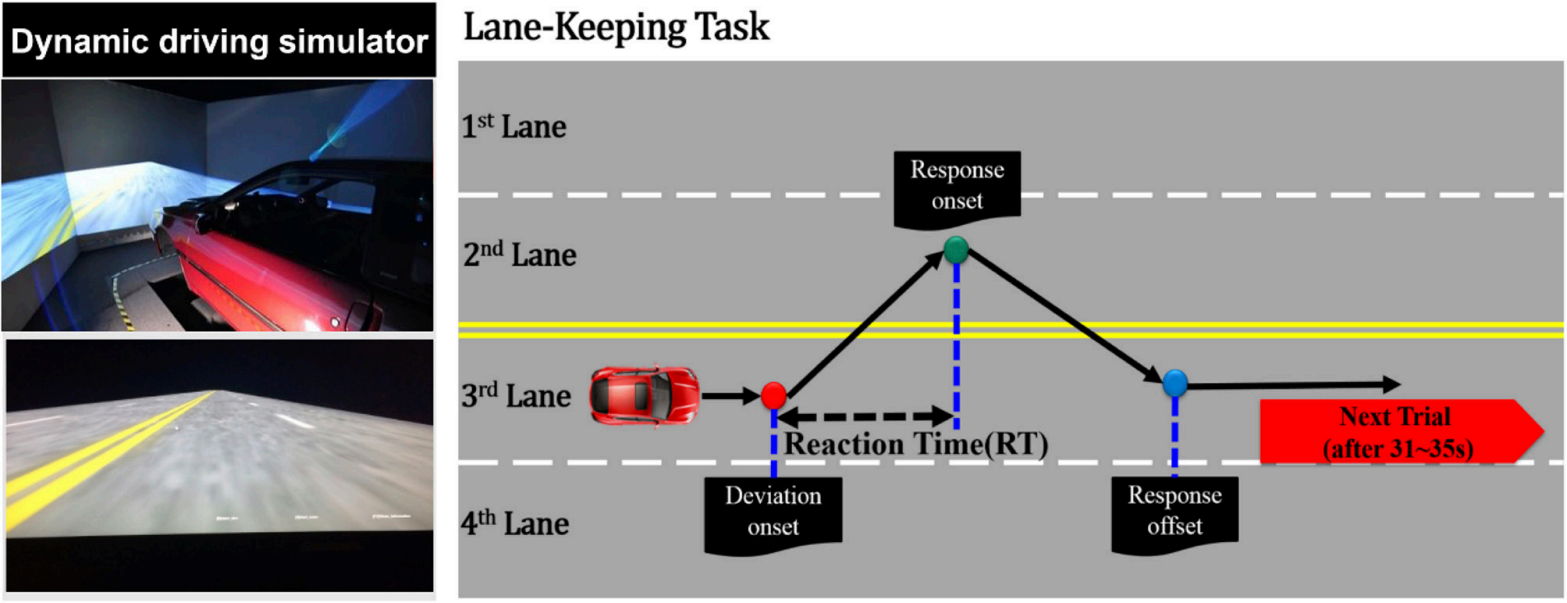
Simulated driving experiment, where the right panel represents the dynamic driving simulator and the left panel represents the event-related lane-departure driving paradigm.

The event-related lane-departure driving paradigm (Huang et al., 2009) was implemented in a virtual driving environment, which was composed of a 360° surrounded scene and a driving simulator (Figure 1). Surrounded scenes comprised six monitors that were controlled through six desktop computers. The driving simulator was a real vehicle equipped with a six degree-of-freedom motion platform. All computers and fictitious driving simulators were connected for the synchronous fictitious driving environment through a Local Area Network.

In this study, the driving scene was an endless four-lane road displayed on the surrounding wall. During the 1-h consecutive driving simulation, the virtual reality scenes simulated driving a vehicle at a specific speed of 100 km per hour on the highway. As shown in Figure 1, a trial is defined as the simulation vehicle randomly drifted away from the center cruising lane (3rd lane) to the right (4th lane), or left (2nd lane) cruising lanes. When the vehicle started to drift out of the center lane, the subjects were required to maneuver the vehicle back to the original lane (3rd lane) and compensate for the drift of the vehicle. The drift inter-trial was set to 31–35 s intervals. In an hour-long driving experiment, there were ~90–110 drift trials in total. The experiment was conducted at approximately noon to match with our previous work (Lin et al., 2013; Chuang et al., 2014; Huang et al., 2016). Before this 1-h driving experiment, the subjects were instructed to maintain alertness and concentrate on the task, even if they felt fatigue.

**Figure 2:**
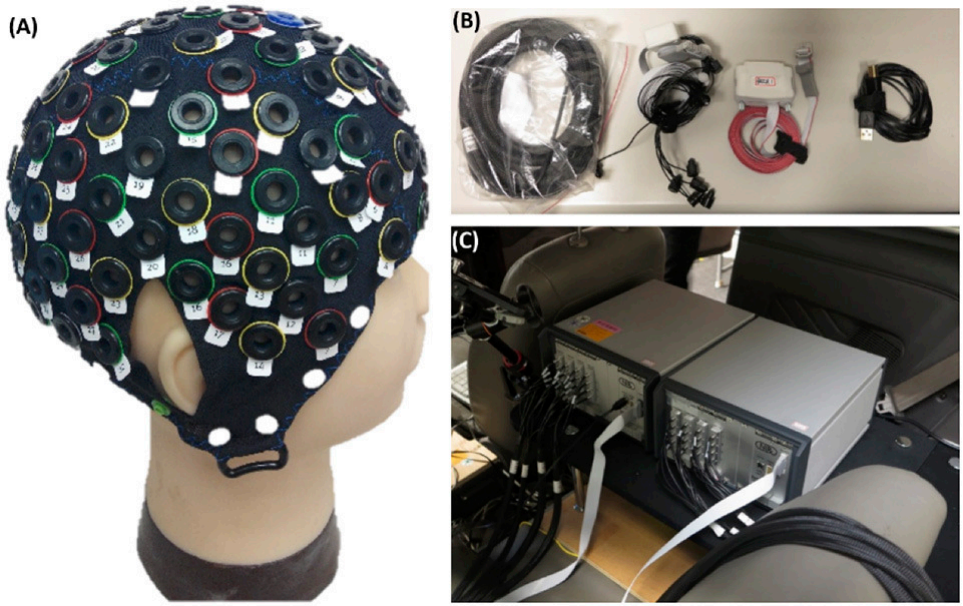
Equipment related to this experiment. **(A)** EEG-fNIRS cap. **(B,C)** fNIRS recording instrument.

At the beginning of the experiment, each subject who wore a suitable cap for recording of the physiological data required 5–10 min to read the experimental instructions and complete the participant information sheet (questionnaire). The noninvasive fNIRS/EEG cap merged functional near-infrared spectroscopic with electroencephalographical sensors (Figure 2). The subjects were seated on a chair to set up the probes of the 8-source and 16-detector NIRS prior to setting conductive gel for EEG data recording. The subjects were simultaneously required to practice the driving task and become familiar with the fictitious driving environments. Moreover, the subjects were required to perform the lane-keeping task during the 1-h (60-min) consecutive driving experiment and pay attention to the deviation situations throughout the experiment. When a deviation occurred, the subjects were required to steer the vehicle back to the cruising lane as fast as possible. After the vehicle returned to the original lane (3rd lane), the next deviation would occur in the following 31–35 s. Of note, the RT, the time duration between the deviation onset and response onset, is recognized to estimate the behavioral performance of drivers.

When an event emerged from the event-related lane-departure driving paradigm, the EEG and fNIRS acquisition system could record three particular points, including the deviation onset, response onset, and response offset. The deviation onset was the moment the vehicle drifted out from the original lane to the other lanes. The response onset was depicted as the time when the subjects began to turn the wheel to the cruising lane to correct the steering and make the vehicle return back to the original lane. The response offset represented the time point at which the vehicle was driven back to the original lane at the scene of the driving experiment and the participant sitting in the simulated vehicle ceased to steer the wheel.

### Simultaneous physiological signal acquisition

#### EEG signal acquisition

The EEG signals were recorded using a Synamp2 system with a 32-Channel Ag/AgCl electrode EEG cap (Compumedical NeuroScan). There were 30 channels located on the cap and two reference channels bipolar attached in both the left and right mastoid bones. All channels were digitized by a 3D-digitizer with the aim of the acquisition of a precise electrode scalp for each participant. The placement of electrodes in the EEG cap was arranged according to a modified international 10–20 system. The skin behind the auricle of the ear, under the reference electrodes, was wiped gently by the examiner using alcohol swabs and nuprep skin prep gel prior to EEG machine calibration. The impedance of each electrode was calibrated under 5 kΩ through NaCl-based conductive gel (Quick-Gel, Neuromedical Supplies). The EEG signals from the electro-cap were amplified and recorded with 32-bit Analog to Digital Converter precision at a sampling rate of 1,000 Hz.

#### fNIR signal acquisition

**Figure 3:**
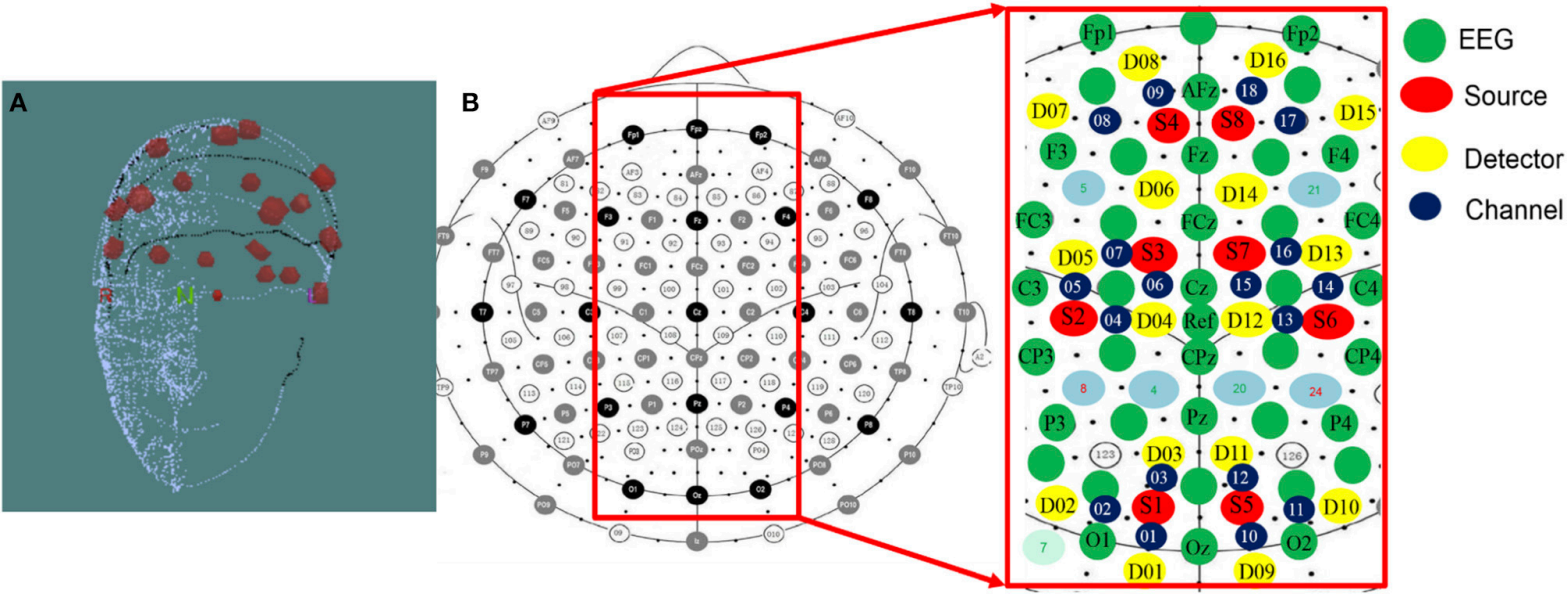
**(A)** 3D Digitizer and **(B)** EEG-NIRS channel location. The 3D Digitizing electrode positions are used to input the three-dimensional head shape. The EEG/fNIRS cap merged functional near-infrared spectroscopic sensors and sources with electroencephalographic sensors. The NIRS optical fibers were positioned 3–4 cm in the proximity of each fiber. The average distance was set approximately 3 centimeters between each source and detector pair, and the measurable depth was 2–3 cm from the scalp.

The NIRScout system (http://nirx.net/nirscout/), from NIRx Medical Technologies, comprised the fNIRS recording platform, which measured the variation of hemodynamic neuro-activation via oxy-, deoxy-, and total- hemoglobin in the cerebral cortex. The NIRScout platform was composed of an 8-source and 16-detector and recorded at a sampling rate of 7.81 Hz based on hybrid EEG-fNIRS studies (Li et al., 2017; Zich et al., 2017). The superficial diffusing probe of the source-detector was at the source wavelengths of 750 and 860 nm, and the inter-optode distance between the source and detector was a maximum of 3.0 cm and a minimum of 2.0 cm. There were 18 channels, as shown in Figure 3, divided into two hemispheres of the cerebral cortex, including the right and left hemispheres.

### Behavioral data analysis

The behavioral data of each participant were recorded using the World Tool Kit (WTK) program. All behavioral data were processed and analyzed via MATLAB software (The Mathworks, Inc.). The RT, the duration time between the deviation onset and response onset, was recorded in each trial throughout the driving experiment by the WTK program. Of note, to ensure the collection of valid behavior data from each subject, the meaningless trials during this experiment (e.g., the driver did not respond to the deviation or the driver responded to the deviation, but shifted into the inverse opposite lane) were removed in the further behavioral analysis. A valid trial occurred when the subject was able to detect the deviation of the vehicle sooner and then turn the steering wheel in the direction opposite to its drifting.

Furthermore, the individual differences of behavior performance among subjects are necessary to take into consideration in our study. To reduce the individual differences in the behavior data (RTs), we used a normalization measurement that subtracted the maximum value of the fastest 10% of RTs from the average value of RTs for each subject.

### EEG data analysis

All EEG data were analyzed using EEGLAB (Delorme and Makeig, 2004), an open-source MATLAB toolbox that supports electrophysiological signal processing, artifact signal rejection, Independent Component Analysis (ICA), time/frequency analysis, statistical analysis, and visualization.

#### EEG preprocessing

The raw EEG signals were preprocessed by 1-Hz high-pass and 50-Hz low-pass finite impulse response (FIR) filters and subsequently down-sampled to 250 Hz to reduce the number of data.

For the artifact rejection, the eye contaminations in EEG signals were manually removed by visual inspection. ICA was subsequently applied to the EEG signals, and the components responsible for the eye movements and blinks were rejected. Moreover, the EEG signals without these artifact components were reconstructed using the back-projection method.

In each trial, the corresponding epoch comprised three periods, which include (1) the baseline period, referred to as the pre-stimulus period (the 1-s period before the onset of the deviation); (2) the lane-departure and steering period (between the onset of the deviation and the end of the response); and (3) the post-movement period (the 5-s period after the end of the response).

#### Independent component analysis

ICA was applied to the EEG signals to separate out temporally independent time courses of the activation (Makeig et al., 1996; Delorme and Makeig, 2004). For each recorded datum, a maximum of 30 independent components (ICs) and the corresponding mixing matrix were decomposed. Of note, the criteria of ICA convergence was set to the maximum steps−1024 and error < 10^7^.

#### Power spectra estimation

Processed data were transformed into the frequency domain by using a 256-point fast Fourier transform with Welch's method. The transformed data were used to estimate of the power spectra with 60 frequency bins from 1 to 30 Hz. The EEG power spectral was separated into the four frequency bands (Delta: 1–3 Hz; Theta: 4–7 Hz; Alpha: 8–12 Hz; Beta: 13–30 Hz). Furthermore, the baseline (pre-stimulus) period of EEG power was used to compare with the event-related EEG power dynamics. Specifically, the average values of the baseline EEG power were subtracted from the event-related EEG power in each epoch, which is directed to diminish the individual difference. The moving-average technique was subsequently used to smooth the event-related time-frequency information to acquire the event-related EEG power spectral.

Furthermore, all frequency responses of EEG activations were calculated using a 512-point moving window without overlapping points. To avoid the confounding issue of classifying unbalanced numbers of effective and ineffective trial samples and the small sample size, a bootstrap cross-validation method (Fu et al., 2005) with 1,000 repeats was performed to bootstrap datasets in specific frequency bins, and then the above procedure was run 30 times in total to test the statistical significance of the EEG power changes from 1 to 30 Hz frequency bins.

### fNIRS data analysis

The fNIR data recording through the NIRSout system was preprocessed and analyzed using the nirsLAB toolbox (Xu et al., 2014), which was executed within the MATLAB environment. The nirsLAB toolbox was based on a graphical user interface for MATLAB, and its resources were mainly used for preprocessing and basic analysis, such as functions for data reading, event editing, filtering, data conversion, and plotting data on a brain map. The function, termed data conversion, converted the amplitude of the original NIRS data from each channel into the level of HbO and HbR concentrations using the familiar modified Beer-Lambert Law (Baker et al., 2014). In general, hemoglobin could be roughly divided into HbO and HbR. HbO is a type of hemoglobin in which the protein molecule in the red blood cells binds to oxygen; in contrast, HbR is the type of hemoglobin that is not bound to oxygen. In our study, the NIRS system includes 8 sources and 16 detectors, which were used to establish the source-detector pairs to acquire fNIRS signals in 18 channels (6 channels in the occipital and parietal areas, 4 channels in the right motor and central areas, 4 channels in the left motor and central areas, and 4 channels in the frontal area).

#### fNIRS preprocessing

We initially used a low-pass filter with a cut-off frequency at 0.4 Hz to remove noise and a high-pass filter of 0.025 Hz to remove the Direct Current. The filtered data were subsequently transferred from ray absorption into HbO and HbR, and the concentration variations in HbO and HbR were calculated by using a modified Beer-Lambert Law (Delpy et al., 1988). This study used the region analysis and averaged the fNIRS information of the same brain region.

#### Event-related fNIRS analysis

In line with the EEG data analysis, the fNIRS data were transformed into the frequency domain and estimate of the power spectra. Furthermore, the baseline (pre-stimulus) period of HbO power was used to compare with the event-related HbO power dynamics. Specifically, the average values of the baseline HbO power were subtracted from the event-related HbO power in each epoch, which is directed to diminish the individual difference. Then, the moving-average technique was used to smooth the event-related fNIRS information and reduce the noise effects.

### EEG-based fatigue levels and normalization

**Figure 4:**
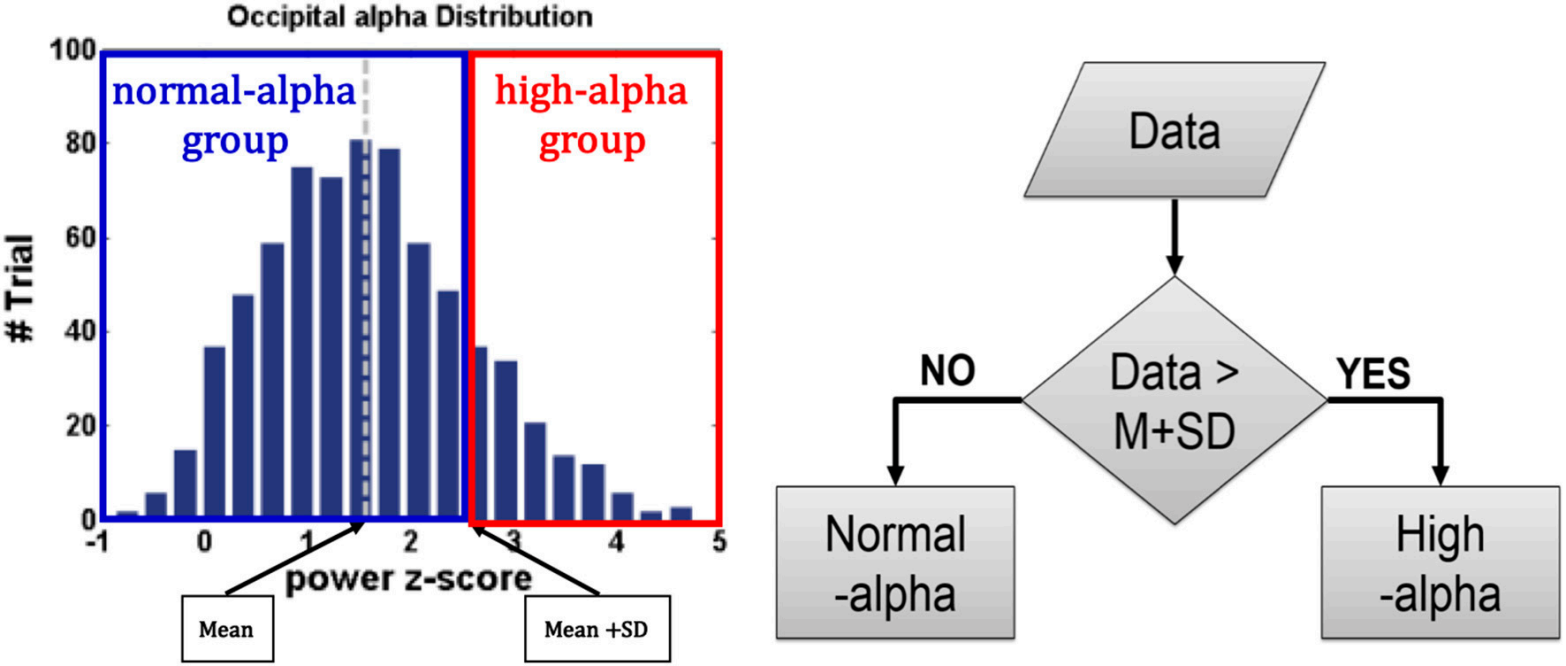
Normalization of EEG-based Fatigue Levels.

For EEG signals of interest, increases in the alpha power were identified as the driving error increased (Campagne et al., 2004) or fatigue occurred (Eoh et al., 2005; Otmani et al., 2005; Jap et al., 2009). The researchers Huang et al. (2009) and Chuang et al. (2014) in our team have reported that the independent EEG components that correspond to the frontal, central, right motor, left motor, parietal, and occipital regions are highly correlated with fatigue, drowsiness, and behavioral lapse, which demonstrated the EEG dynamics have higher reliability than the behavioral performance (e.g., RT). For example, even if drivers experience high fatigue, they can maintain a good behavioral performance. As shown in Figure 4, to recognize EEG-based fatigue levels, the EEG occipital alpha power at baseline was divided into two groups: normal-alpha and high-alpha groups based on the participants by the z-score normalized measurement (*z* = (*x* – μ) / σ, where *x*: the original EEG power; μ: the mean (M) of the EEG power; and σ: the standard deviation (SD) of the EEG power). The normal-alpha group and the high-alpha group are defined as the *z*-scored power < μ + σ and *z*-scored power > μ + σ, respectively.

### Statistical analysis

In this study, a measure of the Pearson correlation was conducted between the EEG frequency power and the behavioral performance (RTs). The Wilcoxon signed-rank test was subsequently used to compare the EEG or fNIRS data between the pre-stimulus and event-related periods. Furthermore, the Wilcoxon rank-sum test was used to compare the event-related EEG or fNIRS data between the normal- and high-alpha groups in different brain regions. The significance level was set at *p* < 0.05. The Pearson correlation was performed in the SPSS software package (version 15.0), and other statistical analysis were conducted in the MATLAB (2011a) Bioinformatics Toolbox.

## Results

### Behavioral performance

**Figure 5:**
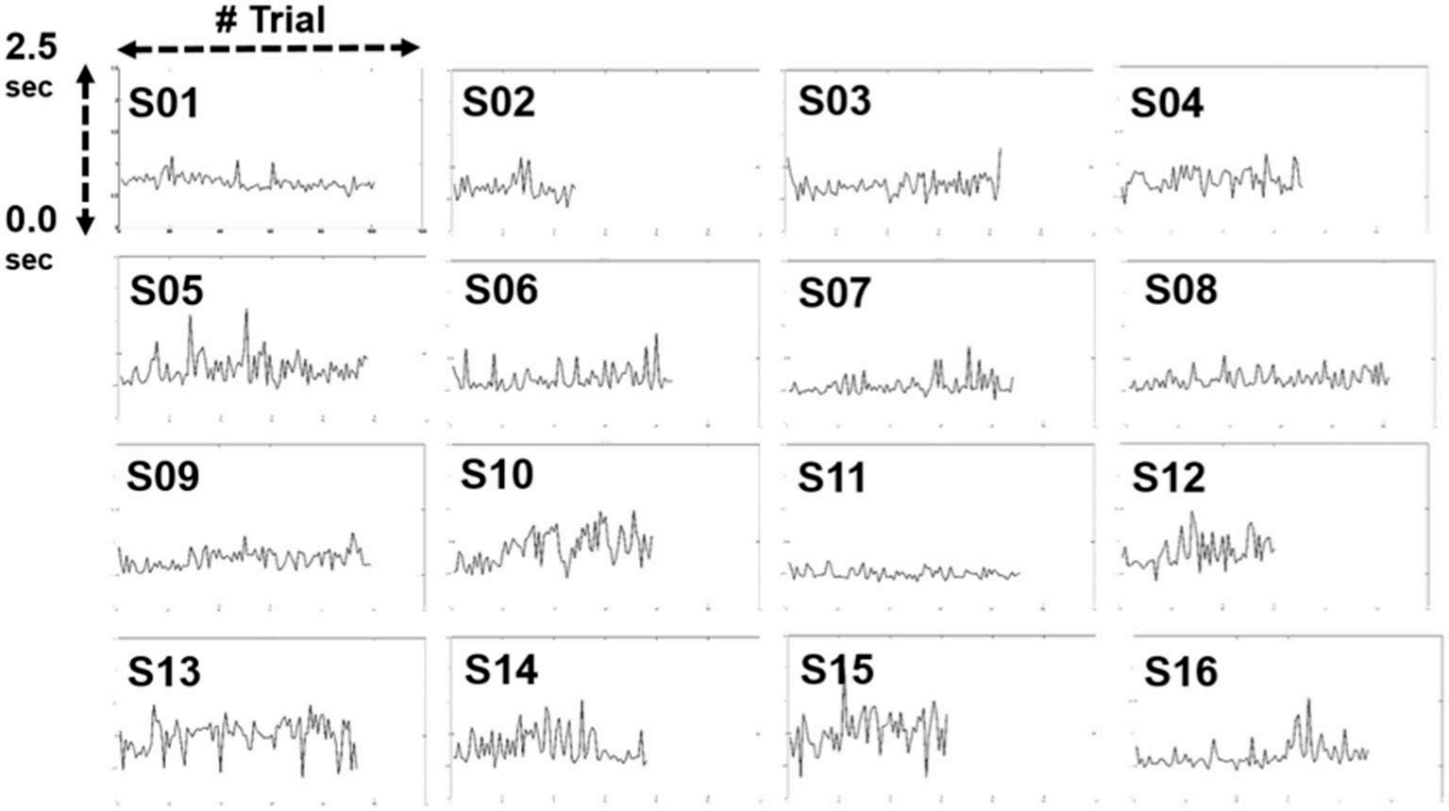
Changes in reaction time in individuals.

Figure 5 shows the changes in the RT across 16 subjects in the 1-h driving task (vertical axis - RT; horizontal axis - numbers of trials). The range of the RT is from 0.0 to 2.5 s. We compared the difference between the first 10% of the trials and the last 10% of the trials during the experiment. The results indicated there was no significant difference in the RT between these two periods (*p*-value > 0.05). Most subjects performed relatively short RTs, which indicates that subjects are able to respond to lane-deviation events in a short time and maintain high attention in an hour-long task.

**Figure 6:**
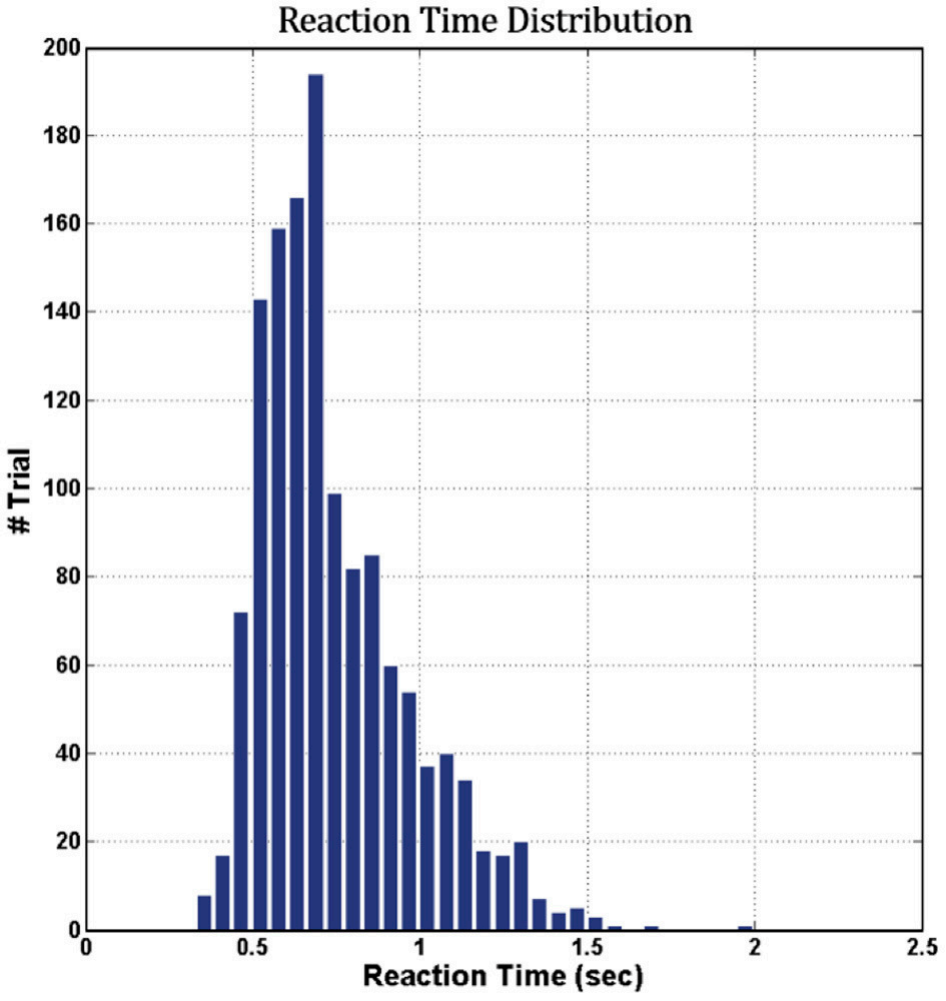
Cumulative distribution of RTs across 16 subjects.

Figure 6 shows the cumulative distribution of RTs across 16 subjects (vertical axis, cumulative number of trials; horizontal axis, corresponding RT in seconds). The range of RTs obtained in this study was from 0.0 to 2.5 s, indicating that all subjects maintained a high task performance on the hour-long task.

### EEG results

**Figure 7:**
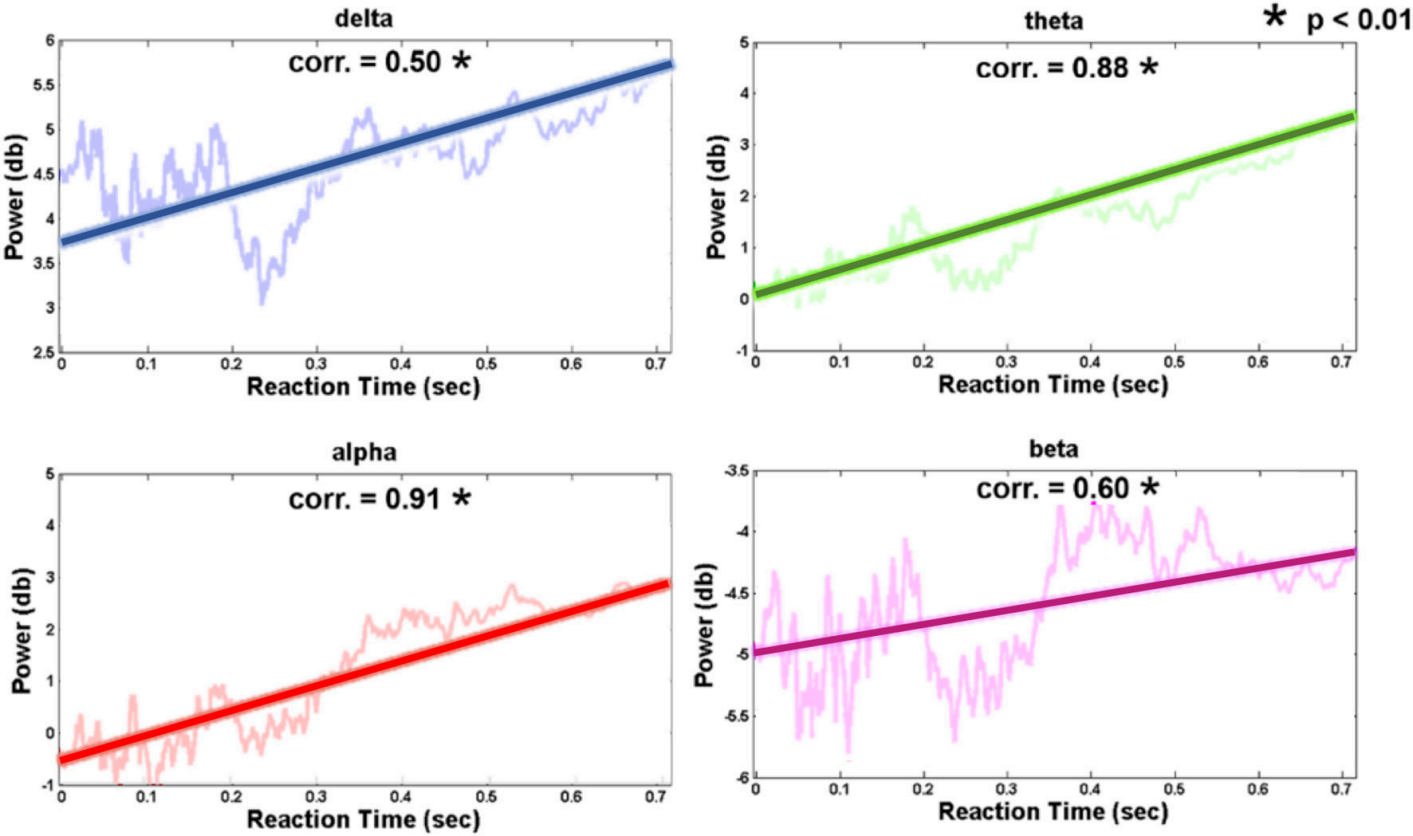
Trends of occipital channel (Oz, O1, and O2) pre-stimulus EEG power in four frequency bands (delta: 1–3 Hz; theta: 4–7 Hz; alpha: 8-12 Hz; beta: 13–30 Hz). The thick and transparent lines represent the regression trend and actual graph in four frequency bands, respectively.

Figure 7 shows the relation between the pre-stimulus occipital EEG log power and RT (vertical axis, power in dB; horizontal axis, RT-sorted index and the corresponding RT). The increased occipital pre-stimulus power was identified in the theta and alpha bands as the RT increased. The correlation coefficients of the RT and pre-stimulus EEG log power were Pearson's *r* = 0.88 (theta power, *p*-value < 0.01) and Pearson's *r* = 0.91 (alpha power, *p*-value < 0.01), which suggests an accumulated fatigue during the driving task. Associated with the behavioral performance, this finding indicated that the participants correctly performed the driving task against cumulative fatigue.

### fNIRS results

**Figure 8:**
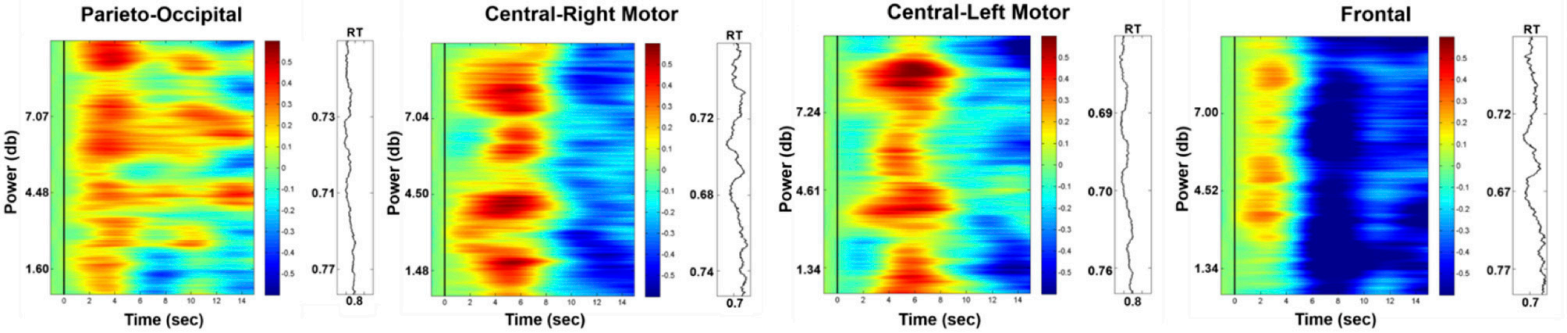
Phasic changes in HbO in parieto-occipital, right motor, left motor, and frontal regions (left vertical axis: EEG power in dB; right vertical axis: corresponding RT; horizontal axis: time in seconds; solid black line: stimulus onset). The red or blue color represent the significant difference (*p*-value < 0.05) between the baseline and deviation periods, and the green color represents no significant difference between these periods.

Figure 8 presents the HbO power changes in the parieto-occipital, right motor, left motor and frontal regions, respectively. The results demonstrated significant increases in the HbO power of the deviation periods in these four brain regions compared to the baseline (*p*-value < 0.05). This enhanced HbO power is consistent with the increased EEG alpha power at the occipital region.

### Fatigue levels: RT vs. EEG

**Figure 9:**
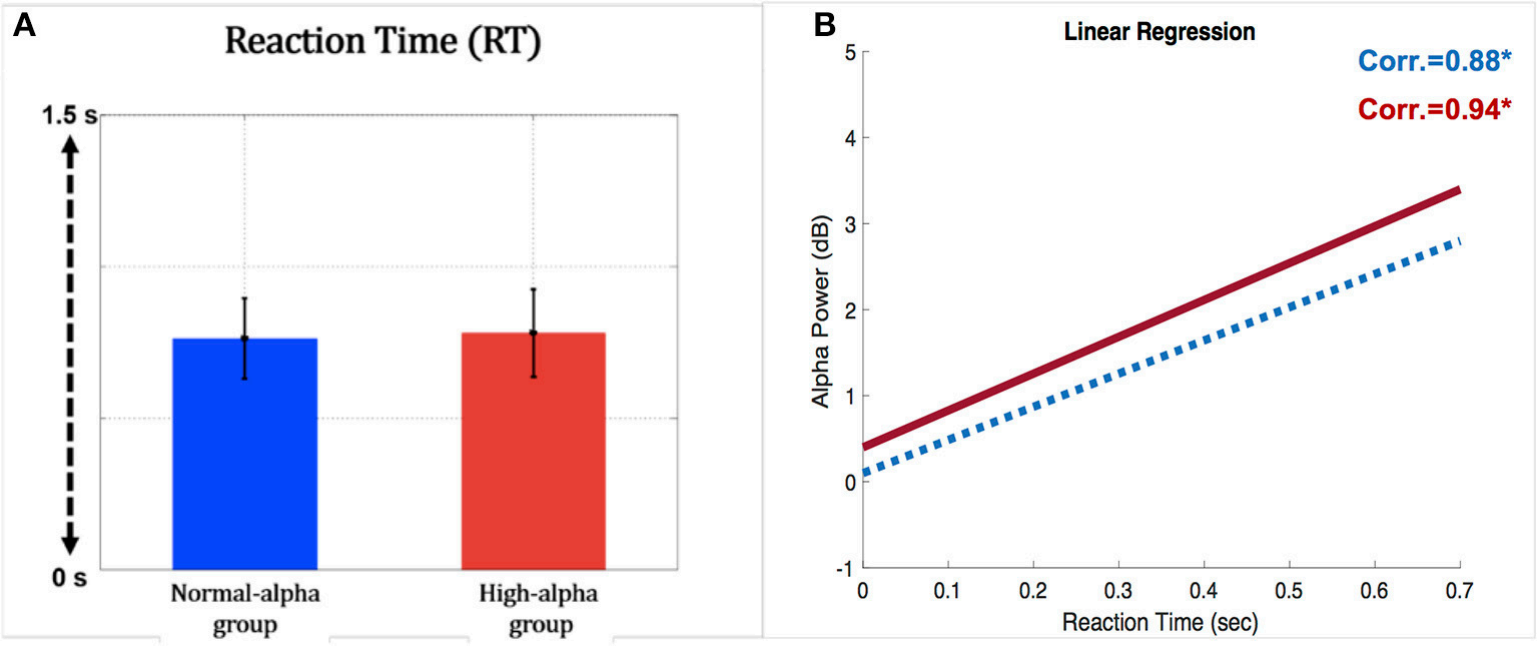
**(A)** Comparison of reaction time for the occipital pre-stimulus alpha power (vertical axis: RT (second); horizontal axis; blue bar: normal-alpha group; red bar: high-alpha group); **(B)** correlation between the RT and EEG alpha power (horizontal axis: RT (second); vertical axis: power (dB); blue line: normal-alpha group; red line: high-alpha group).

EEG and fNIRS were divided into two groups (high alpha vs. normal alpha) based on the pre-stimulus occipital alpha power, which represents the participants' levels of fatigue and driving performance. Figure 9A presents the comparison of task performance between the normal-alpha group and high-alpha group. The results showed that there is no significant difference (*p*-value = 0.63, *Z*-value = 1.79) between the groups.

Furthermore, the normal-alpha group and high-alpha group are compared together for the occipital pre-stimulus alpha power. The high-alpha group has higher occipital pre-stimulus alpha power than normal-alpha group (*p*-value = 0.0005, *Z*-value = 3.06). Based on Figure 7, we calculated the correlation (*r*) between the RT and EEG alpha power in the normal-alpha group and high-alpha group, respectively. As shown in Figure 9B, the results showed that *r* = 0.88 (*p*-value = 0.01, *Z*-value = 2.87), and *r* = 0.94 (*p*-value = 0.03, *Z*-value = 2.54) in the normal-alpha group and high-alpha group, respectively. This separate result is consistent with the mixed results for two groups (section EEG Results).

### Hemodynamic correlates of EEG changes

**Figure 10:**
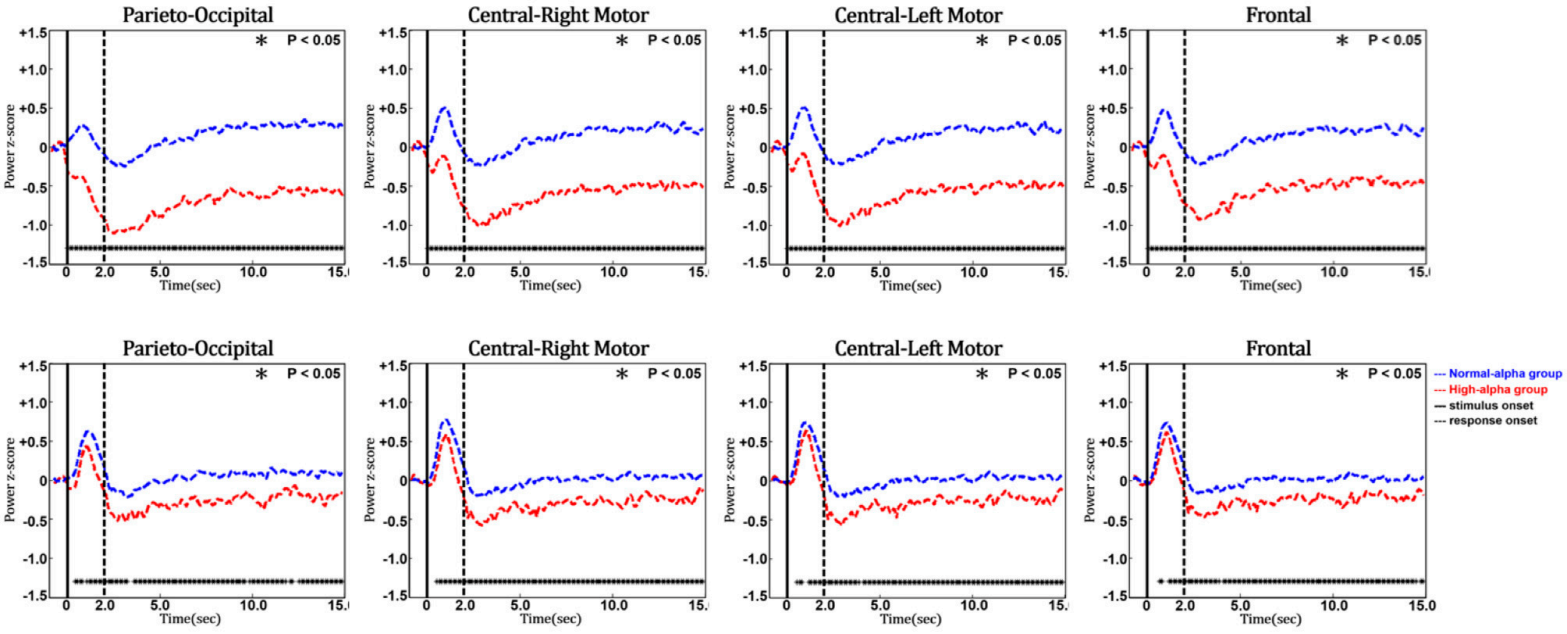
Comparison of event-related EEG alpha (upper panel) and theta (lower panel) dynamics between normal- and high-alpha groups in different regions (vertical axis: EEG power in z-score; horizontal axis: time in seconds; solid line: stimulus onset; dashed line: response onset).

Figure 10 shows the phasic changes of the EEG alpha and theta powers in the parieto-occipital, central-right motor, central-left motor, and frontal regions. Both the alpha and theta powers exhibited a significant pulse immediately after stimulus onset (*p*-value < 0.05), which was followed by a decrease after the response onset in four selected regions. The statistical analysis further indicated that there were significant differences in the phasic EEG alpha and theta powers between the normal-alpha group and high-alpha group.

**Figure 11:**
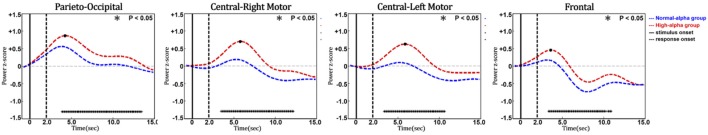
Phasic changes of oxygenated hemoglobin in different regions (vertical axis: power in z-score; horizontal axis: time in seconds; solid line: stimulus onset; dashed line: response onset).

Figure 11 shows the phasic curve of HbO in the parieto-occipital, central-right motor, central-left motor, and frontal regions. The results indicated that there were significant differences in the changes in the phasic HbO power between the normal-alpha group and high-alpha group after the response onset (*p*-value < 0.05) in these four selected regions. In addition, HbO increased when the pre-stimulus EEG alpha power increased in the occipital region, which indicates that the corresponding region requires more oxygen consumption when the fatigue level increases during this experiment. Of note, there is no significant difference between the normal-alpha and high-alpha groups during the latency period.

Furthermore, a *t*-test's effect size (*d*) indicates whether there is a difference in the EEG or HbO power between the two groups (normal-alpha vs. high-alpha) on a continuous dependent time variable. Our results showed *d* > 1.16 when the brain dynamics showed the significant difference between the two groups (*p*-value < 0.05).

## Discussion

This study investigated the effect of cumulative fatigue on simulated driving using an event-related lane-departure paradigm and simultaneous EEG-fNIRS recordings. Recently, hybrid approaches have combined two different modalities to investigate brain signatures and improve performance. In this study, we simultaneously record fNIRS and EEG during a fatigue-fighting driving experiment and subsequently distinguish drivers with high- and low-levels of fatigue with neuro-physiological correlates. Of note, the fNIRS system measures hemodynamic change, which is a delayed response compared to neuronal electrical activity, and it also has a relatively low temporal resolution (<10 Hz), both of which are critical drawbacks in fNIRS measurements.

In general, driving performance has represented an index of mental fatigue in some studies. However, it does not fit the fatigue-fighting study because subjects have to maintain their RT in event-related lane-departure during an hour-long experiment. Furthermore, mental fatigue was related to increases in the pre-stimulus EEG alpha band in the occipital region (Huang et al., 2009), which is regarded as a reliable indicator to recognize fatigue levels.

We used the occipital (Oz, O1, O2) pre-stimulus EEG alpha power to divide the phasic data into two groups, which included the normal-alpha group and high-alpha group. Based on these two groups, we observed the phasic changes in HbO and HbR in certain brain regions for further discoveries. The phasic analysis showed HbO. Moreover, the latencies of the peak activation for HbO in all regions are different, particularly in the right and left motor regions, which are longer than the parieto-occipital and frontal regions.

Driving performance together with EEG alpha powers have comprised a commonly used index to measure mental fatigue (Huang et al., 2009; Wascher et al., 2014). Previous studies have shown that the increased alpha power was observed as the driving error increased (Campagne et al., 2004) or fatigue occurred (Eoh et al., 2005; Otmani et al., 2005; Jap et al., 2009). As shown in Figure 7, our finding demonstrated that the increasing pre-stimulus EEG alpha power was accompanied by an increasing RT (correlation *r* = 0.91), which suggests accumulated fatigue during the experiment. In terms of behavioral performance (RT), each participant can respond to lane-deviation events in the short time. Thus, although participants showed the accumulated fatigue, they can adjust for the deviation in a timely manner. These findings indicated that participants had to correctly perform the task against cumulative fatigue, and we referred to this phenomenon as fatigue fighting in driving.

In this study, subjects were instructed to maintain their task performance as much as they could throughout the experiment, which indicates that the subjects had to perform the task against cumulative fatigue. The findings showed that fatigue-fighting episodes occurred in the trials with high alpha powers. Although the sign of fatigue occurs in the brain, all participants are able to respond to lane-deviation events immediately, which suggests that they are fighting fatigue during the experiment. The event-related analysis further shows that strengthening alpha suppression in the occipital region and increasing HbO in the frontal cortex, supplementary motor area, primary motor cortex, and parieto-occipital cortex indicate that the subjects are fighting fatigue.

There was no significant difference in cerebral HbR. In recent years, numerous researchers have attempted to identify the physiological relations between neuronal alpha oscillations and cortical activations through combined analysis of EEG and fMRI. Based on previous EEG-fMRI related studies, EEG alpha activity is negatively correlated with the blood-oxygen-level dependent (BOLD) signal over the frontal, parietal, and occipital areas in the resting state (Goldman et al., 2002; Laufs et al., 2003; Gonçalves et al., 2006; Michels et al., 2010; Scheeringa et al., 2012). Moreover, there are contrary relations between the EEG alpha power and BOLD signals in the occipital areas; thus, EEG alpha amplitudes are related to decreases in BOLD signals in the visual cortex (Moosmann et al., 2003; Feige et al., 2005).

In contrast to EEG, fNIR is also used to monitor cerebral blood oxygenation during this fatigue-fighting driving task. Previous studies have shown a significant reduction in the cerebral oxygen saturation when a driver's performance deteriorated (Li et al., 2009) and no significant change in the cerebral oxygen exchange in the prefrontal cortex during constant velocity driving on an expressway (Yoshino et al., 2013). In this study, we determined that it is important to observe the increases in cerebral HbO at several seconds after the stimulus event among all related regions in the normal-alpha and high-alpha groups. Our findings showed that strengthening alpha suppression in the occipital region and increasing HbO in the frontal cortex, supplementary motor area, primary motor cortex, and parieto-occipital cortex indicate that subjects are fighting fatigue.

However, there are several limitations in this study. First, we performed only an off-line analysis. Therefore, real-time data analysis must be developed to apply a combined EEG and NIRS system in actual driving conditions. Second, we need to collect additional EEG and NIRS data to confirm our preliminary results. Third, the demonstrated results combined EEG/fNIR data, and it remains unclear whether it is possible to predict driver fatigue in advance to prevent motor vehicle collapse. Finally, the error or missed trials are considered ineffective trials in this study because participants failed to react against the accumulated fatigue in these periods. However, these ineffective trials also include significant information to identify EEG dynamics in a high fatigue state.

## Conclusions

This study investigates the physiological phenomena in response to a lane-departure driving task with simultaneous measurement of EEG-fNIRS. We observe the relation between the level of mental fatigue and brain dynamic activities in an event-related paradigm during a 1-h driving experiment. The results show that significant differences are observed in the EEG alpha power in the occipital regions, which is a commonly used indicator of fatigue. All participants may become fatigued gradually, but they are able to respond to lane-deviation events immediately during this driving task. The event-related analysis further shows strengthening alpha suppression in the occipital region, which indicates that participants have to exert more effort to maintain attention. In addition, HbO increases in the phasic aspect in the frontal cortex, supplementary motor area, primary motor cortex, and parieto-occipital cortex. Taken together, all increased neural activity and cortical activations are observed in drivers to maintain task performance when fatigue occurs. The electrodynamic and hemodynamic signatures of fatigue fighting provided in this study might improve the understanding of the neural mechanism of fatigue.

## Author contributions

C-HC, J-TK, and C-TL: Designed the research plan and organized the study; C-HC, ZC, J-TK, B-SW, and Y-KW: Coordinated the data analysis and interpreted the data; C-HC and ZC: Wrote the manuscript; B-SW: Administered the experiments. All authors discussed the results and commented on the manuscript.

### Conflict of interest statement

The authors declare that the research was conducted in the absence of any commercial or financial relationships that could be construed as a potential conflict of interest.
